# GENES FROM PEROXISOME AND INTEGRATED STRESS RESPONSE IN REGULATION OF HUMAN LONGEVITY

**DOI:** 10.1093/geroni/igad104.2490

**Published:** 2023-12-21

**Authors:** Anatoliy Yashin, Deqing Wu, Konstantin Arbeev, Hongzhe Duan, Igor Akushevich, Arseniy Yashkin, Rachel Holmes, Svetlana Ukraintseva

**Affiliations:** Social Science Research Institute, Duke University, Durham, North Carolina, United States; Social Science Research Institute, Duke University, Durham, North Carolina, United States; Social Science Research Institute, Duke University, Durham, North Carolina, United States; Social Science Research Institute, Duke University, Durham, North Carolina, United States; Duke University, Durham, North Carolina, United States; Duke University, Durham, North Carolina, United States; Social Science Research Institute, Duke University, Durham, North Carolina, United States; Duke University, Durham, North Carolina, United States

## Abstract

Dietary/caloric restriction shares pathways with peroxisome, a cellular organelle that regulates the synthesis and turnover of complex lipids. PEX3, a peroxisomal biogenesis factor involved in selective type of autophagy. EIF2AK4, a sensor of amino acid deprivation in the integrated stress response (ISR) involved in lifespan regulation. We hypothesize that interplay between EIF2AK4 and PEX3 is associated with human survival. We applied logistic regression model with the interaction term to the analysis of Health and Retirement Study (HRS) and UK Biobank (UKB) parents data to test whether interactions of SNPs from the EIF2AK4 and SNPs from the PEX3 are associated with the dichotomous survival trait (ST) with case group defined as: lifespan ≥ 85; and control as: 75 ≤ (lifespan or age at last follow-up) < 85. Analyses of HRS data showed that interactions between SNPs rs4924405, rs2250402, rs8031319, rs16970049, rs3816899, rs16970112, rs2412459 from the EIF2AK4 gene and SNP rs161070 from the PEX3 gene are significantly associated with ST. In the UKB data, interactions of rs161070 from the PEX3 gene and rs7176881 and rs117778159 SNPs from the EIF2AK4 gene showed nominally significant associations with ST. Analyses of linkage disequilibrium (LD) between the EIF2AK4 SNPs detected in HRS and UKB data showed that their LD are close to the highest values for SNPs with allele frequencies specific for detected variants. Our results confirm association of interplay between EIF2AK4 and PEX3 with human longevity. They also show how information from experimental studies could be transformed to the form useful for human applications.

